# Supplementary material for: A semi-synthetic regulon enables rapid growth of yeast on xylose
Source: Nat Commun. 2018 Mar 26;9:1233. doi: 10.1038/s41467-018-03645-7 (PMC5964326; doi:10.1038/s41467-018-03645-7)
Supplement: Supplementary file 1 — Supplementary Information(PDF 1565 kb) [file 41467_2018_3645_MOESM1_ESM.pdf]

A semi-synthetic regulon enables rapid growth of yeast on xylose

Endalur Gopinarayanan et al.

Suplemental Note 1: Characterization of expression strengths of GAL-activated versus constitutive promoters

To assess which Gal4p-mediated genes assist in growth on galactose, we compared growth between strains that have Leloir genes under GAL-activated promoters (WT strain, GAL-REG) and constitutive promoters (GAL-CONS). First, we compared the expression strengths of GAL-activated (*GAL1p*, *GAL7p*, and *GAL10p*) and constitutive promoters (*TEF1p*, *TPI1p*, and *GPM1p*) by placing EGFP gene downstream of these promoters. Since the WT strain has a single copy of Leloir pathway genes, we placed the $GAL_{promoter}$ – EGFP constructs in pRS406 integration plasmid and knocked it into the *URA3* locus of the chromosome. To account for locus-based expression differences, we also cloned the constructs in low copy plasmids (2 – 5 copy number[1]) and considered them to be the maximum possible expression of GAL promoters in the WT strain. Next, we cloned EGFP under constitutive promoters in multicopy pRS426 plasmid and compared their fluorescence in galactose **(Supplementary Fig.1a).** Comparing the results, we observe that the constitutive promoters in high copy plasmids have stronger expression strength than single copy GAL promoters integrated in the chromosome or similar expression strength with low-copy GAL promoters. This observation is consistent with mRNA expression data obained from RNA-seq analysis **(Supplementary Fig.1b)**.

Supplementary Note 2: Engineering Gal3p mutants for better binding to xylose

The fluorescence profile of Gal3p titrated with varying concentrations of xylose suggested that Gal3p can accommodate xylose **(Fig. 3B)**. Further, it is known that upon binding to its ligand, Gal3p undergoes a conformational change that enables it to dimerize with, and sequester, Gal80p and activate the GAL regulon. Since Gal3p-Gal80p interaction is the key step involved in activation of the GAL regulatory system, we decided to strengthen its interaction by mutagenizing Gal3p[2]. Analysis of the Gal3p-Gal80p[3] co-crystal structure revealed two loops on Gal3p that interacts with Gal80p **(Supplementary Fig.2A)**. Residues from 93-115 (referred as loop 1) form a dynamic loop that remains closed when Gal3p is in *apo*-form but opens up during Gal3p-galactose interaction. A second stationary loop (loop 2) from 345-381 also interacts with Gal80p. We carried out random mutagenesis using error-prone PCR (epPCR) on the two loops, to obtain a mutant library of 10^4 variants with loops 1 and 2 having an average of 1.2 ± 0.8 and 1.1 ± 0.5 amino acid mutations respectively. After selection and screening with 2 % xylose, we obtained a mutant, Gal3p-1.1, which had a significantly higher fluorescence in xylose than Gal3p-WT **(Supplementary Fig.2B)**. Next, we characterized the fluorescence profile of the mutant by varying the concentration of xylose from 4 % to 0.002 % and observed more than 7-fold increase in fluorescence above 2 % xylose concentration **(Fig. 3C)**. Fluorescence dropped down significantly at xylose concentrations below 0.2 % and was indistinguishable from the control, confirming that the mutation did not result in constitutive activation of the GAL regulon. We sequenced the mutant and found it to be an A109T mutation. Analysis of the Gal3p crystal structure shows A109 residue present on the dynamic loop of Gal3p facing Gal80p **(Supplementary Fig.2A).** When Gal3p-1.1 was titrated with different concentrations of galactose, we observed a 100-fold increase in sensitivity to galactose as well **(Fig. 3D)**. Taken together, results indicate that the mutation seems to increase Gal3p-Gal80p interaction efficiency resulting in increased sensitivity and fluorescence upon induction with xylose and galactose.

To explore other residues at position 109 that could improve fluorescence, we carried out single site saturation mutagenesis with NNK codons to obtain a diversity of 32 codons and screened 3,000 variants (~100-fold coverage). The fluorescence profile of the best mutant, Gal3p-2.1 (A109V mutation), indicated a marked increase in signal strength and sensitivity at 2 % and 0.2 % xylose. In fact, there was an almost twelve-fold change in fluorescence at 2 % xylose (**Fig. 3C**). Simultaneously, we also explored mutations that could further increase the GAL regulon induction strength through epPCR based random mutagenesis on the entire protein with Gal3p-1.1 as the template. From a library of 10^5 variants with an amino acid mutation rate of 1.9 ± 0.8, through selection and screening, we obtained three variants with better fluorescence profiles **(Supplementary Fig.2B)**. All three variants showed increased signal strengths. Gal3p-3.1 and Gal3p-3.3 exhibited ten-fold increase in signal at 2 % xylose. Gal3p-3.2 had much lower

fold induction values due to higher background fluorescence **(Fig. 3C)**. Although the fold change varied between the mutants, the fluorescence at 2 % xylose concentration were relatively similar and comparable to Gal3p-2.1 mutant. However, the fold increase in fluorescence at 0.2 % xylose was less than five-fold in these mutants. Previous studies on xylose metabolism in *S. cerevisiae* have suggested transport as a rate limiting step at low xylose concentrations[4]. To alleviate any possible xylose transport limitations, we knocked-in an engineered xylose transporter *GAL2-2.1*[5,6] at the *LEU* locus of VEG16 to create VEG20, which was used for the next round of mutagenesis.

Sequencing of the Gal3p-3.1, Gal3p-3.2, and Gal3p-3.3 mutants revealed that some of the mutations introduced during random mutagenesis arose close to one another **(Supplementary Table 3)**. We employed a primer-based synthetic shuffling strategy[7] to combine mutations using multiple primers containing degenerate codons that coded for wild-type or the mutated nucleotides. We amplified and spliced together six fragments with *GAL3-2.1* as the template to cover the eight mutations from variants 3.1, 3.2, and 3.3 and transformed VEG20 with the library for functional selection. Since the motivation to use synthetic shuffling was to obtain mutants with better sensitivity, we carried out selection and screening at 0.2 % xylose. The best mutant, Gal3p-Syn4.1 showed a fluorescence profile with lower background, higher sensitivity, and a ten-fold increase in fluorescence at 0.2 % xylose **(Fig. 3C)**.

Supplementary Note 3: ODE models for single and dual feedback systems:

The feedback models were adapted from Venturelli et al.[8] with modifications as described in the methods section, including all of the parameters used **(Supplementary Table 4)**. Fluorescence assay for measuring Gal3p-WT-galactose-Gal80p interaction was compared with the model prediction. While dual and single feedback showed a difference as predicted by the model, the trends were different. To match them, the forward binding rate constant, kf83, was decreased to 2.5 $nM^{-1}min^{-1}$. Further, it is known that Gal4p binds cooperatively to UAS_{GAL} sites present on GAL activated promoters[9,10]. We checked the cooperativity of *GAL10p, GAL3p* and *GAL80p* promoters used in the model experimentally by using the promoters to drive EGFP and measured fluorescence output after inducing them with varying concentrations of xylose **(Supplementary Fig.3)**. We fit the data to Hill equation and obtained cooperativity values of 2, 1, and 1 for *GAL10p*, *GAL3p*, and *GAL80p* respectively, which were used for both the feedback models. It is to be noted that the parameter kf83 has been chosen to better reflect the experimental data and that the conclusion drawn from the experiments does not change when kf83 is modified in the model, provided the order of binding interaction strength for the three cases are preserved: Gal3pWT-xylose-Gal80p < Gal3p-Syn4.1-xylose-Gal80p < Gal3p-WT-galactose-Gal80p. The simulations along with experimental data confirm that while at low interaction strengths Gal3p-sugar interaction is inefficient, as the interaction gets stronger, the difference between the two models becomes more prominent with the dual feedback showing increased signal.

Single feedback model:

$$\frac{dG3}{dt} = vgal + \alpha G3 * \frac{[G4]^{n3}}{KG3^{n3} + [G4]^{n3}} - kf83\,[G3][G80] + kr83[C83] - \gamma_{G3}\,[G3]$$

$$\frac{dG4}{dt} = \alpha G4 - kf84\,[G4][G80] - \gamma_{G4}[G4]$$

$$\frac{dG80}{dt} = \alpha_o G80 + \alpha G80 * \frac{[G4]^{n80}}{KG80^{n80} + [G4]^{n80}} - kf83\,[G3][G80] + kr83[C83] - \gamma_{G80}\,[G80] - kf84\,[G3][G80] + kr83[C84] - \gamma_{G80}\,[G80]$$

$$\frac{dGFP}{dt} = \alpha G1 * \frac{[G4]^{n1}}{KG1^{n1} + [G4]^{n1}}$$

$$\frac{dC83}{dt} = kf83\,[G3][G80] - kr83[C83] - \gamma_{C83}[C83]$$

$$\frac{dC84}{dt} = \; kf84\,[G4][G80] - kr84[C84] - \; \gamma_{C84}[C84]$$

Dual feedback model:

$$\frac{dG3}{dt} = vgal + \; \alpha G3 * \frac{[G4]^{n3}}{KG3^{n3} + \; [G4]^{n3}} + \; \alpha G1 * \frac{[G4]^{n1}}{KG3^{n1} + \; [G4]^{n1}} - kf83\,[G3][G80] + kr83[C83] - \; \gamma_{G3}\,[G3]$$

$$\frac{dG4}{dt} = \alpha G4 - kf84\,[G4][G80] - \; \gamma_{G4}[G4]$$

$$\frac{dG80}{dt} = \alpha_o G80 + \; \alpha G80 * \frac{[G4]^{n80}}{KG80^{n80} + [G4]^{n80}} - kf83\,[G3][G80] + kr83[C83] - \; \gamma_{G80}\,[G80] \; - kf84\,[G3][G80] + kr83[C84] - \; \gamma_{G80}\,[G80]$$

$$\frac{dGFP}{dt} = \alpha G1 * \frac{[G4]^{n1}}{KG1^{n1} + \; [G4]^{n1}}$$

$$\frac{dC83}{dt} = \; kf83\,[G3][G80] - kr83[C83] - \; \gamma_{C83}[C83]$$

$$\frac{dC84}{dt} = \; kf84\,[G4][G80] - kr84[C84] - \; \gamma_{C84}[C84]$$

Supplementary Note 4: Activation of downstream genes under the synthetic xylose regulon

It has been shown previously that the GAL regulon differentially regulates hundreds of genes which include not only genes of galactose metabolism (*GAL1*, *GAL7*, *GAL10*) and regulation (*GAL3*, *GAL80*, *GAL4*) but also genes responsible for other cellular functions such as *GCY1, FUR4, LAP3, MTH1, PCL10, REE1,* etc[11–13]. By amplifying the upstream regions of genes *GCY1*, *LAP3*, *MTH1*, *PCL10*, *FUR4*, and *REE1,* and placing them upstream of *EGFP* we created *Promoter-EGFP-TEFt* constructs. As control, we also included the constitutive promoter *TEF1p* promoter upstream of *EGFP-TEFt*. The constructs were grown in sucrose and tested for fluorescence with or without the regulon. As positive control, the strains were also incubated in galactose, to check the maximum possible induction for these promoters. **Supplementary Fig.6** shows the fluorescence obtained under the conditions tested. Only three of the six promoters, *GCY1p*, *MTH1p*, and *PCL10p* had increased fluorescence with the xylose regulon consistent with fluorescence increase observed when the same genes were induced with galactose. Under these assay conditions, we did not observe activation of the other three promoters even though it has been shown to be upregulated by galactose[13,14]. To check if there is sucrose induced regulation of these genes, we switched the growth medium from sucrose to a mixture of ethanol (3 %) and glycerol (2 %). With the new growth medium, we observed activation of *LAP3p* by both xylose and galactose, suggesting possible sucrose-mediated regulation of *LAP3p*. The other two promoters, *FUR4p* and *REE1p*, probably have weak up-regulation that couldn't be detected by the fluorescence assay. Overall, we show that in the presence of xylose, Gal3p-Syn4.1 controls genes of the GAL regulon that are both strongly and weakly *trans*-activated, similar to galactose-based activation **(Fig. 4G)**.

Supplementary Note 5: Characterization of expression strengths of *GAL1p/10p* and constitutive promoters in xylose

To check if there is a difference in expression strengths between *GAL1p/10p* and *TEF1p/TPI1p* results in observed difference in growth rates of XYL-REG and XYL-CONS strain in xylose, we transformed VEG16 strain with either pRS426-GAL1p, pRS426-GAL10p, pRS426-TEF1p or pRS426-TPI1p plasmid along with XYL regulon plasmids (pVEG16* and pVEG17*) and compared the fluorescence levels after growing the strains in sucrose along with xylose. To replicate the conditions of growth on xylose, we used high copy plasmids. Since XYL-REG and XYL-CONS strains were grown in 2 % xylose, we checked for fluorescence in 2 % xylose, as well at maximum xylose concentration of 4 %. From **Supplementary Fig.7a**, it can be seen that the fluorescence at 2 % xylose concentration, constitutive promoters have 1.6-fold higher fluorescence than the GAL promoters and at 4 % xylose concentration, the fluorescence levels remain the same. On the contrary, mRNA expression data from RNA-seq analysis reveal mRNA levels of *XYLA*3* and *XKS1* from XYL-REG to be several folds higher than XYL-CONS **(Supplementary Fig.7b)**. It is possible that either increased levels of mRNA doesn't translate completely into protein or presence of sucrose as the growth substrate suppresses GAL regulon resulting in lowered fluorescence of strains carrying pRS426-GAL1p and pRS426-GAL10p plasmids. Either way, the difference in expression strengths of GAL regulated promoters and constitutive promoters alone does not explain the difference in growth rates as increased expression need not directly translate to increased growth rate. In the case of GAL-CONS, though expression of *GAL1,7,10* genes were several folds higher than in GAL-REG, growth rate was lower. Finally, as seen from RNA-seq analysis **(Fig. 6)**, the increased expression of XYLA*3 and XKS1 in XYL-REG is not sufficient to explain the observed upregulation of several growth-related pathways.

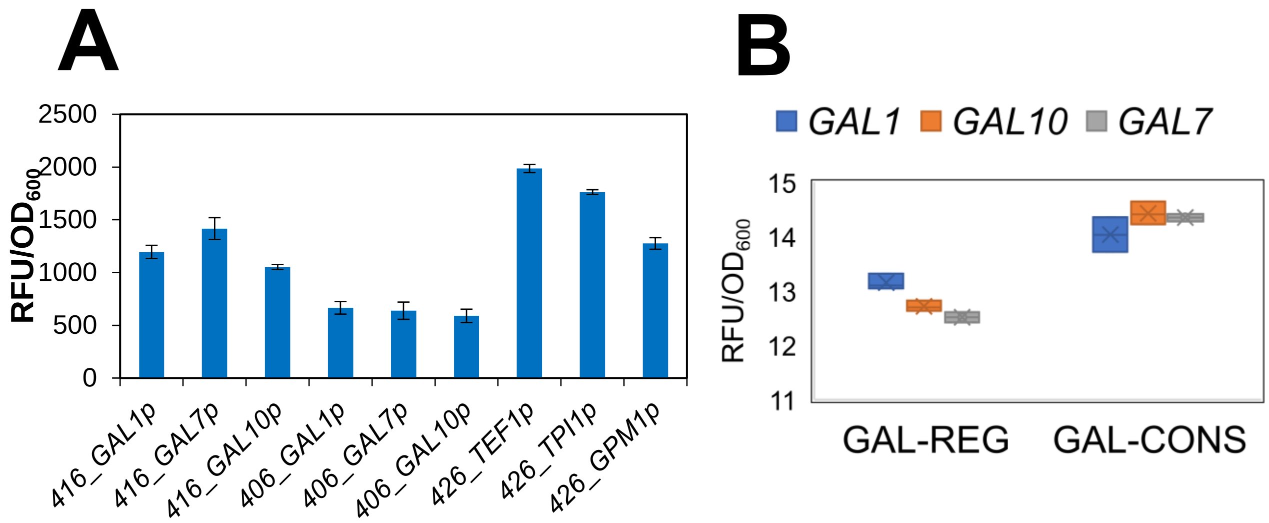

Supplementary Figure 1: Comparison of expression strengths of GAL regulated and constitutive promoters when grown in galactose. (A) GAL and constitutitve promoters expressing EGFP in single chromosomal copy (406), low copy plasmid (416), and multicopy plasmid (426). (B) Box and whisker plot of normalized log counts per million mapped reads of *GAL1*, *GAL7*, and *GAL10* mRNA in GAL-REG (WT) and GAL-CONS strains. Each data point represents average of three biological replicates ± sd.

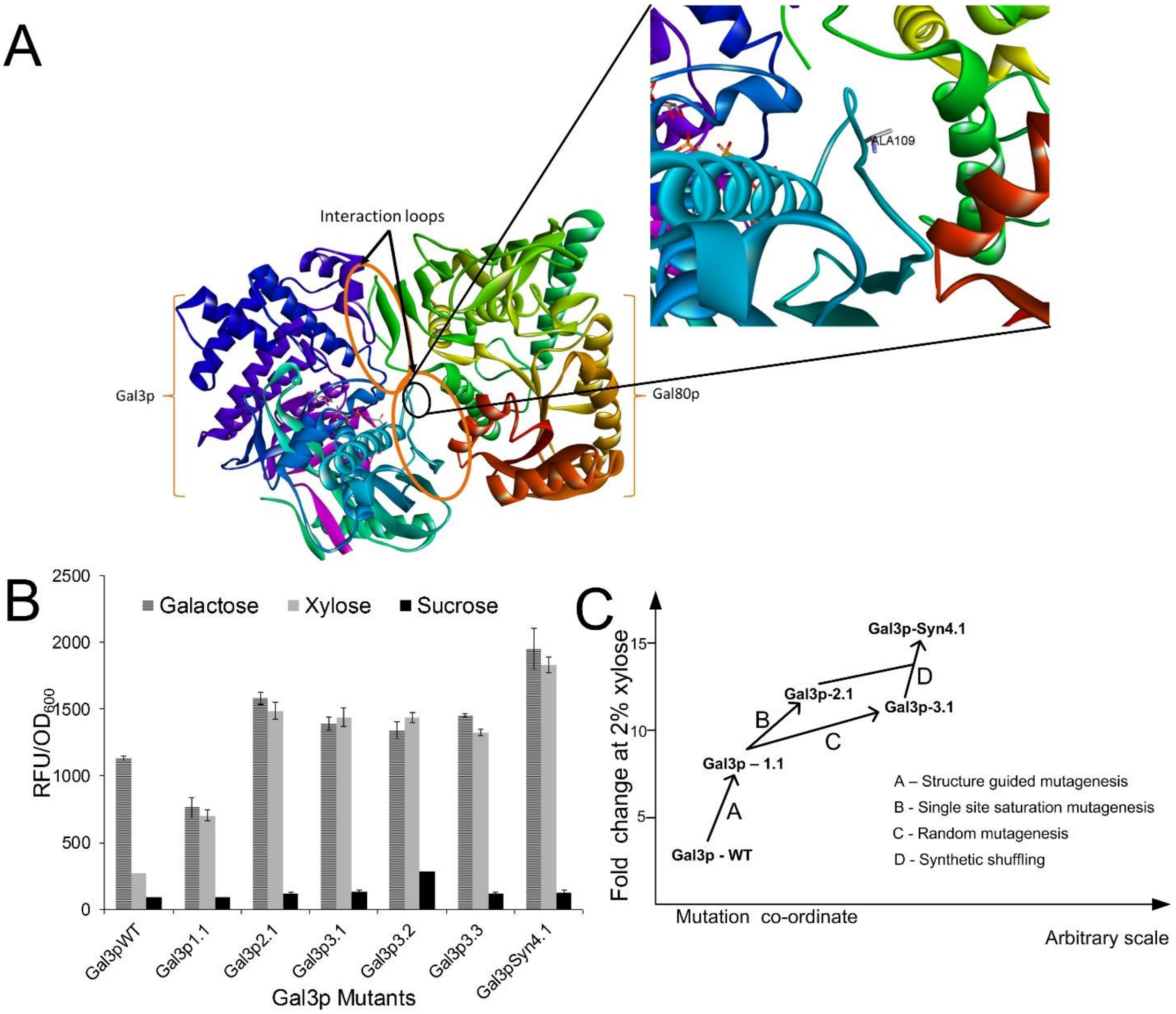

Supplementary Figure 2: Gal3p-mutagenesis. (A) Crystal structure of Gal3p with the two interaction loops circled in orange (figure adapted from Lavy T, et al., 2012[3]). The position of A109 residue is zoomed in to show that A109 faces the Gal80p suggesting that mutation to Thr or Val probably increases this interaction and thus increases the strength of GAL regulon activation by xylose. (B) Fluorescence of Gal3p-WT and the best mutants from successive rounds of mutagenesis were incubated with sucrose alone, 2 % xylose or 2 % galactose. (C) Roadmap to the final mutant Gal3p-Syn4.1 and increase in fold change at 2 % xylose with every successive round of mutation. Each data point represents average of biological triplicates ± sd.

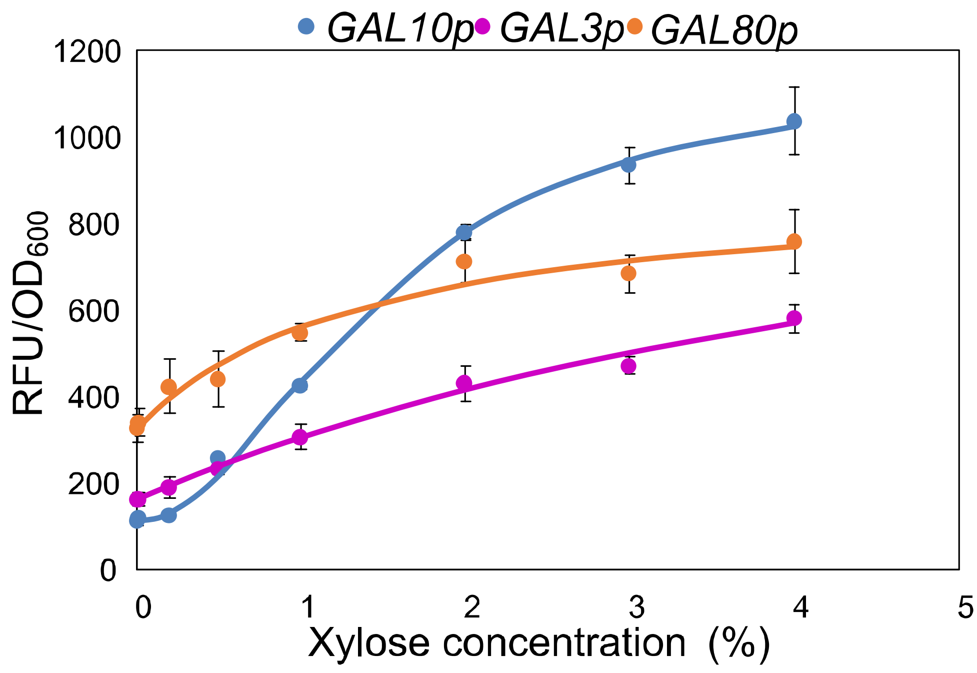

Supplementary Figure 3: Fluorescence assay of constructs with *GAL10p*, *GAL3p*, and *GAL80p* promoters driving EGFP expression. Data points represent actual experiments and the lines represent hill curve fits for the data. Each data point represents average of biological triplicates ± sd.

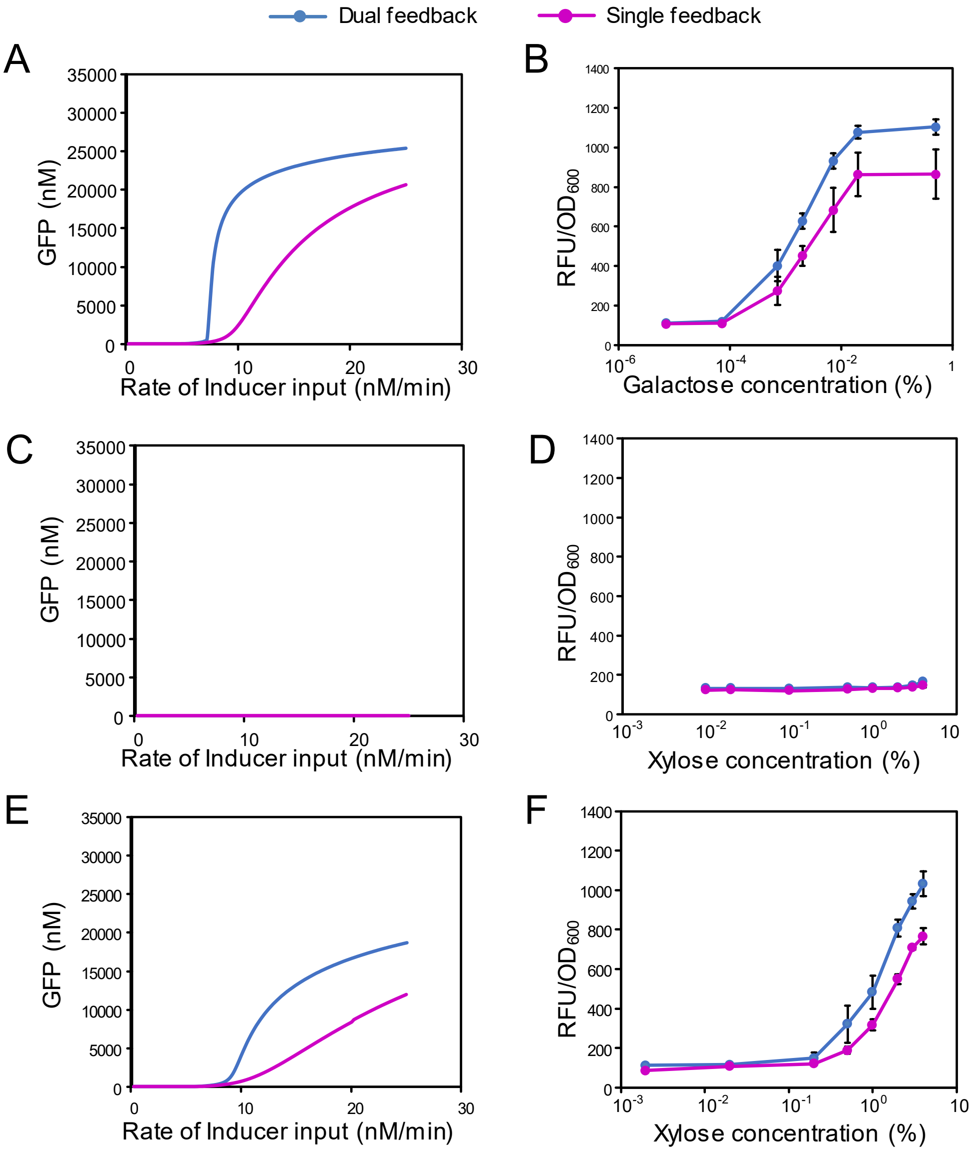

Supplementary Figure 4: Comparison of steady state ODE simulation for single and dual feedback models with varying levels of kf83 with experimental data measuring varying interaction strengths of Gal3p-sugar-Gal80p binding. (A & B) Comparison of simulation and experiment for Gal3p-WT-galactose-Gal80p interaction with the model having kf83 of 2.5. (C & D) Comparison of simulation and experiment for Gal3p-WT-xylose-Gal80p interaction with the model having kf83 of 0.1. (E & F) Comparison of simulation and experiment for Gal3p-Syn4.1-xylose-Gal80p interaction with the model having kf83 of 1. Each data point represents average of biological triplicates ± sd.

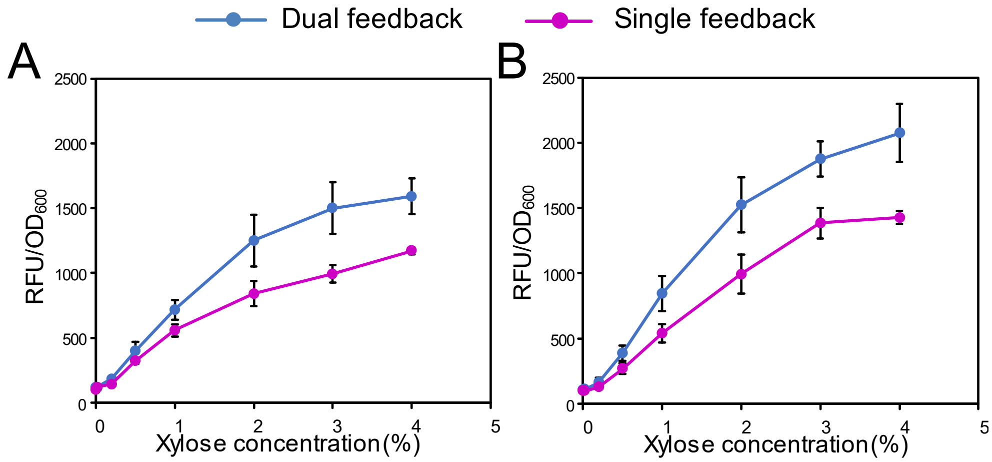

Supplementary Figure 5: Increase in sensitivity of dual positive feedback system over single feedback system using (A) *GAL1P-EGFP-T* and (B) *GAL7P-EGFP-T* as reporter constructs. Each data point represents average of biological triplicates ± sd.

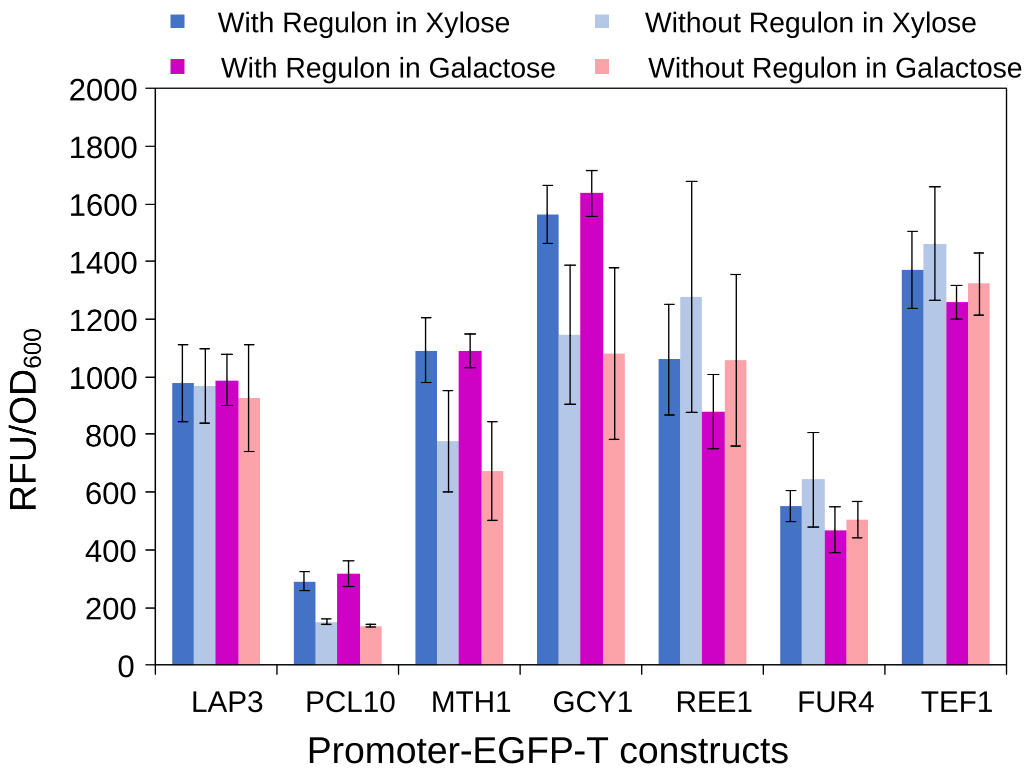

Supplementary Figure 6: Some of the promoters that drive the downstream genes of the galactose regulon were used to drive EGFP. Fluorescence was measured in the presence or absence of xylose and galactose regulon when grown in sucrose. Each data point represents average of biological triplicates ± sd.

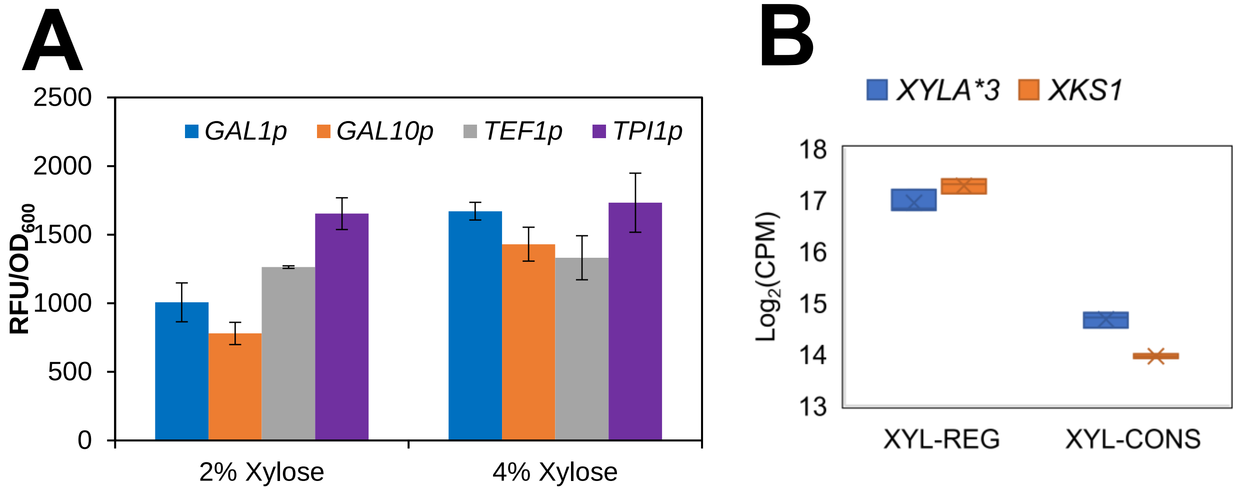

Supplementary Figure 7: Comparison of expression strengths of GAL regulated and constitutive promoters when grown in xylose. (A) GAL and constitutitve promoters expressing EGFP when grown in sucrose with 2 % or 4 % xylose. (B) Box and whisker plot of normalized log counts per million mapped reads of *XYLA** and *XKS1* mRNA in XYL-REG and XYL-CONS strains. Each data point represents average of biological triplicates ± sd.

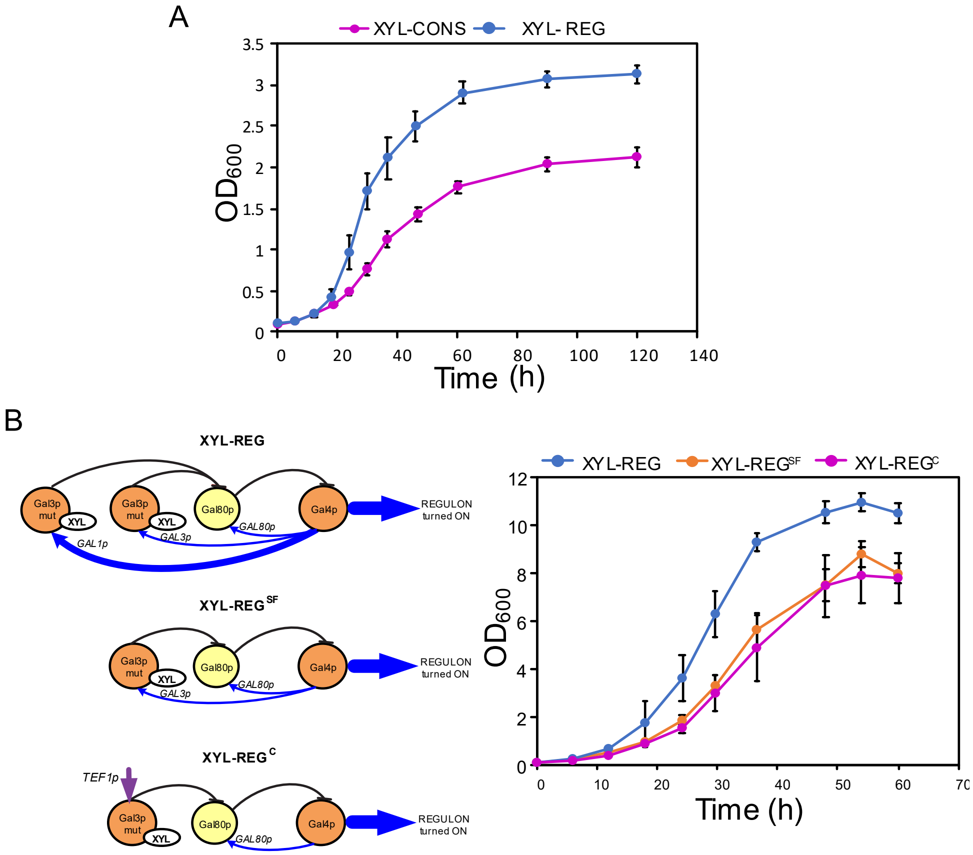

Supplementary Figure 8: Growth of engineered strains in xylose. (A) Growth of XYL-REG (μ = 0.12 h^{-1}, OD_{max} ≈ 3.2) and XYL-CONS (μ = 0.07 h^{-1}, OD_{max} ≈ 2.1) strains in minimal medium supplemented with 2 % xylose. (B) Schematic of the regulon designs (left) and comparison of growth curves (right) in complex medium (YPA + 2% xylose) of dual feedback strain XYL-REG (μ = 0.15 h^{-1}, OD_{max} ≈ 11), with single feedback, XYL-REG^{SF} (μ = 0.12 h^{-1}, OD_{max} ≈ 8), and constitutively active XYL regulon XYL-REG^{C} (μ = 0.12 h^{-1}, OD_{max} ≈ 8). Each data point represents average of biological triplicates ± sd.

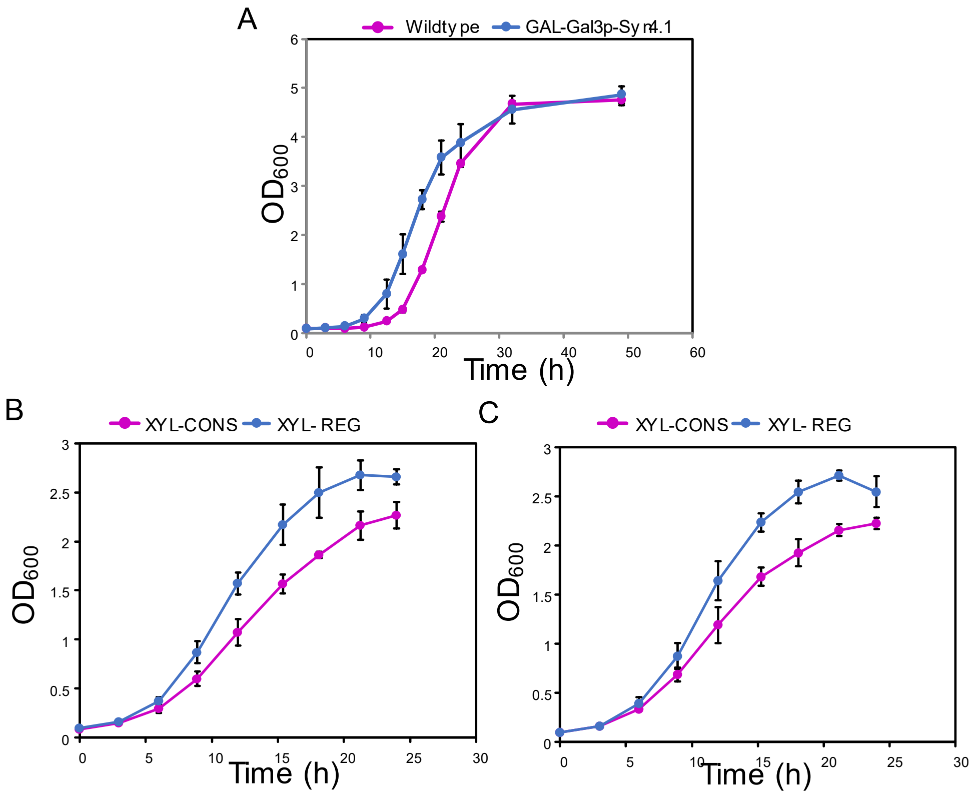

Supplementary Figure 9: Growth of strains in inducing and non-inducing sugars. (A) Growth of wildtype and a strain expressing Gal3p-Syn4.1 in a *GAL3Δ* background on 2 % galactose ($\mu = 0.3\ h^{-1}$ for both). Growth of strains XYL-REG and XYL-CONS in minimal media supplemented with (B) non-repressing sugar sucrose and (C) repressing sugar glucose. Each data point represents average of biological triplicates ± sd.

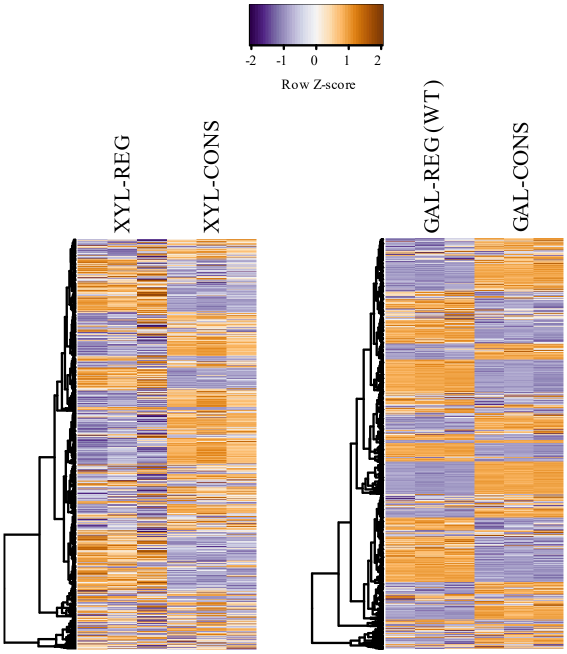

Supplementary Figure 10: Hierarchically clustered heatmap of transcriptome profiles of REG vs CONS strains.

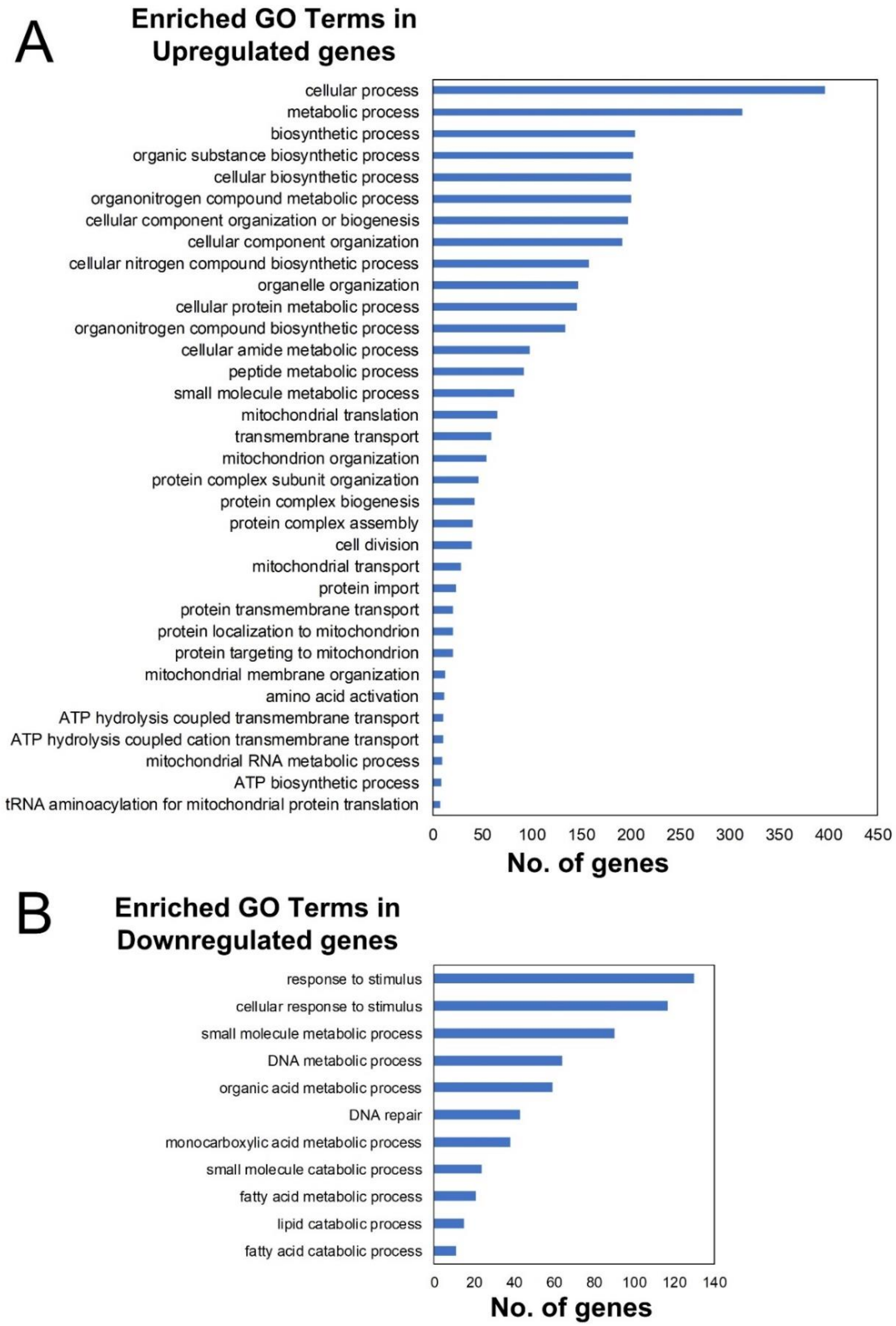

Supplementary Figure 11: GO term enrichment analysis of genes that are (A) upregulated in REG strains (B) downregulated in REG strains, both compared to CONS strains.

Supplementary Table 1: List of mutations on the Gal3p variants during each round of mutagenesis.

**Mutant name**	**Mutations**	**Round**
Gal3p-1.1	A109T	Interaction loop mutagenesis
Gal3p-2.1	A109V	Site Saturation Mutagenesis
Gal3p-3.1	K22R, D68N, I77T, C123G, N141S, L394I, A109T	Error prone PCR
Gal3p-3.2	I271L, L394I, A109T	Error prone PCR
Gal3p-3.3	V69M, L394I, A109T	Error prone PCR
Gal3p-Syn4.1	D68N, V69M, I271L, A109V	Synthetic shuffling

Supplementary Table 2: List of plasmids used.

**Plasmids**	**Description**
**pCONS-GAL**	*pRS426***,** *2µ ori, URA3, TEF1t-GAL7-GPM1p-ADH1t- GAL10- TPI1p-TEF1p –GAL1-HXT7t*
**pVEG7**	*pRS426, 2µ ori, URA3, ADH1t-EGFP- GAL1p/GAL10p-KANMX-Hxt7t*
**pVEG8**	*pRS426, 2µ ori, URA3, GAL3p-GAL3-TEF1t, ADH1t-EGFP- GAL1p/GAL10p-KANMX-HXT7t*
**pVEG11**	*pRS426, 2µ ori, URA3, ADH1t- Piromyces_XYLA*3- GAL1p/GAL10p -XKS1-HXT7t*
**pVEG12**	*pRS423, 2µ ori, HIS, ADH1t- TAL1- GAL1p/GAL10p–GAL2-2.1-HXT7t*
**pVEG13**	*pRS423, 2µ ori, HIS, ADH1t- TAL1- TEF1p-TPI1p –GAL2-2.1-HXT7t*
**pVEG15**	*pRS426, 2µ ori, URA3, ADH1t- Piromyces_XYLA*3- TEF1p-TPI1p -XKS1-HXT7t*
**pVEG16**	*pRS414, CEN ori, TRP, GAL3p-GAL3-TEF1t*
**pVEG17**	*pRS415, CEN ori, LEU, GAL1p-GAL3-TEF1t*
**pVEG16***	*pRS414, CEN ori, TRP, GAL3p-GAL3-Syn4.1-TEF1t*
**pVEG17***	*pRS415, CEN ori, LEU, GAL1p-GAL3-Syn4.1-TEF1t*
**pVEG16**^{C}	*pRS414, CEN ori, TRP, TEF1p-GAL3-Syn4.1-TEF1t*
**pRS405-GAL2-2.1**	*pRS405, no ori, LEU, GAL2p-GAL2-2.1-TEF1t*
**pRS426-GAL1p**	*pRS426, 2µ ori, URA3, GAL1p-EGFP-ADH1t*
**pRS426-GAL3p**	*pRS426, 2µ ori, URA3, GAL3p-EGFP-ADH1t*
**pRS426-GAL80p**	*pRS426, 2µ ori, URA3, GAL80p-EGFP-ADH1t*
**pRS426-GAL7p**	*pRS426, 2µ ori, URA3, GAL7p-EGFP-ADH1t*
**pRS426-GAL10p**	*pRS426, 2µ ori, URA3, GAL10p-EGFP-ADH1t*
**pRS416-GAL1p**	*pRS426, CEN ori, URA3, GAL1p-EGFP-ADH1t*
**pRS416-GAL7p**	*pRS426, CEN ori, URA3, GAL7p-EGFP-ADH1t*
**pRS416-GAL10p**	*pRS426, CEN ori, URA3, GAL10p-EGFP-ADH1t*
**pRS406-GAL1p**	*pRS406, URA3, GAL1p-EGFP-ADH1t*
**pRS406-GAL7p**	*pRS406, URA3, GAL7p-EGFP-ADH1t*
**pRS406-GAL10p**	*pRS406, URA3, GAL10p-EGFP-ADH1t*

**pRS426-REE1p**	*pRS426, 2μ ori, URA3, REE1p-EGFP-ADH1t*
**pRS426-LAP3p**	*pRS426, 2μ ori, URA3, LAP3p-EGFP-ADH1t*
**pRS426-PCL10p**	*pRS426, 2μ ori, URA3, PCL10p-EGFP-ADH1t*
**pRS426-FUR4p**	*pRS426, 2μ ori, URA3, FUR4p-EGFP-ADH1t*
**pRS426-MTH1p**	*pRS426, 2μ ori, URA3, MTH1p-EGFP-ADH1t*
**pRS426-GCY1p**	*pRS426, 2μ ori, URA3, GCY1p-EGFP-ADH1t*
**pRS426-TEF1p**	*pRS426, 2μ ori, URA3, TEF1p-EGFP-ADH1t*
**pRS426-TPI1p**	*pRS426, 2μ ori, URA3, TPI1p-EGFP-ADH1t*
**pRS426-GPM1p**	*pRS426, 2μ ori, URA3, GPM1p-EGFP-ADH1t*

Supplementary Table 3: List of strains used.

Strains	Description
**W303-1a (GAL-REG)**	*MATa leu2-3,112 trp1-1 can1-100 ura3-1 ade2-1 his3-11,15*
**VEG6**	*W303-1a, ΔGAL3*
**VEG16**	*W303-1a, ΔGAL3; ΔGRE3; ΔGAL1; ΔGAL7; ΔGAL10*
**VEG20**	*W303-1a, ΔGAL3; ΔGRE3; ΔGAL1; ΔGAL7; ΔGAL10; LEU::GAL2p-GAL2-2.1-TEFt*
**CONS-GAL**	*W303-1a, ΔGAL4;* transformed with pCONS-GAL
**CONS-GAL-*GAL4***	*W303-1a, ΔGAL3; ΔGRE3; ΔGAL1; ΔGAL7; ΔGAL10;* transformed with pCONS-GAL
**XYL-CONS**	VEG16 transformed with pVEG15, pVE13, pRS41416 and pRS415
**XYL-REG**	VEG16 transformed with pVEG11, pVE12, pVEG16* and pVEG17*
**$XYL\text{-}REG^{SF}$**	VEG16 transformed with pVEG11, pVE12, pVEG16* and pRS415
**$XYL\text{-}REG^{C}$**	VEG16 transformed with pVEG11, pVE12, $pVEG16^{C}$ and pRS415
**GAL-Gal3p-Syn4.1**	VEG6 transformed with $pVEG16^{Syn4.1}$
**VEG21**	*W303-1a, ΔGAL3; ΔGRE3; ΔGAL1; ΔGAL7; ΔGAL10; URA3::GAL10p-EGFP-TEFt*
**VEG22**	*W303-1a, ΔGAL3; ΔGRE3; ΔGAL1; ΔGAL7; ΔGAL10; URA3::GAL1p-EGFP-TEFt*
**VEG23**	*W303-1a, ΔGAL3; ΔGRE3; ΔGAL1; ΔGAL7; ΔGAL10; URA3::GAL7p-EGFP-TEFt*

Supplementary Table 4: List of primers used

Primer Name	Primer Sequence
**Gene deletion primers**	
For-Ura3-Gal4pend	cgatgcacagttgaagtgaacttgcggggtttttcagtatgtttagcttgcctcgtcccc
Rev-Ura3-Gal4pend	gtgcaattaatttttcctattgttacttcgggcctttttccactggatggcggcgttagt
F-Gal4p-Ura3removal	ttgaagtgaacttgcggggtttttcagtatgaaaaaggcccgaagtaacaataggaaaaa
R-Gal4p-Ura3removal	tttttcctattgttacttcgggcctttttcatactgaaaaaccccgcaagttcacttcaa
For-Ga4p-seq	agatcagaggttacatggcc
Rev-Gal4p-seq	tccgcgtcctttgagacagc
Forward Gal3/CaUra3	ttttactgaaacgtatataatcatcataagcgacaagtgagctttctcaggtatagtatg
Reverse Gal3/CaUra3	cttaaaatagctccgcggatgctagatttctacgagtcataactatagggagaccggcag
Forward Gal3/CaUra3 Removal	ttttactgaaacgtatataatcatcataagcgacaagtgatatgactcgt
Reverse Gal3/CaUra3 Removal	cttaaaatagctccgcggatgctagatttctacgagtcatatcacttgtcg
Forward Sequencing Gal3	aaagaatgaaatcgccatgccaagcc
Reverse Sequencing Gal3	taaacggaaatgggcggacattta
Forward Gal1,7,10/CaUra3	caggcattagtgcttttataagcataagaagttgtggcgagtatgaggtcgctcttattg
Reverse Gal1,7,10/CaUra3	tccaaaacgcagcggttgaaagcatatcaagaattttgtcttaatgcaggttaacctggc
Forward Gal1,7,10/CaUra3 Removal	caggcattagtgcttttataagcataagaagttgtggcgagacaaaattc
Reverse Gal1,7,10/CaUra3 Removal	tccaaaacgcagcggttgaaagcatatcaagaattttgtctcgccacaac
Forward Sequencing Gal1,7,10	aacataggtgcaggatttcc
Reverse Sequencing Gal1,7,10	acaaagggttctcgtagagt
Forward GRE3/CaUra3	cgaagttactacttctagggggcctatcaagtaaattactgtatgaggtcgctcttattg
Reverse GRE3/CaUra3	tatacagcatcggaatgagggaaatttgttcatatcgtcgttaatgcaggttaacctggc
Forward GRE3/CaUra3 Removal	cgaagttactacttctagggggcctatcaagtaaattactcgacgatatg
Reverse GRE3/CaUra3 Removal	tatacagcatcggaatgagggaaatttgttcatatcgtcgagtaatttac
Forward Sequencing GRE3	ggattaaaagggagcccaag
Reverse Sequencing GRE3	gtcaaccatacaagagatga
**Primers for pCONS-GAL**	
F-Teft-SacI-pRS426end	tgattacgccaagcgcgcaattaaccctcactaaagggaacaaaagctggagctctggatggcggcgttagtatc
R-Teft-Gal7end	gattcattatctacaaagactgtaagacaataaaaagattcttgttttca
F-Gal7-Teftend	tgaaaacaagaatctttttattgtcttacagtctttgtagataatgaatc
R-Gal7-GPM1end	caaacaaacacacatattacaataatgactgctgaagaatttgatttttc
F-GPM1-Gal7end	gaaaaatcaaattcttcagcagtcattattgtaatatgtgtgtttgtttg
R-GPM1-NotI-ADH1tend	tgaccacacctctaccggcagcggccgctgcaatgtatgactttaagatt
F-ADH1t-NotI-GPM1end	aatcttaaagtcatacattgcagcggccgctgccggtagaggtgtggtca
R-ADH1t-Gal10end	caagattgtctacagattttcctgaggcgcgccacttctaaataagcgaa
F-Gal10-ADH1tend	ttcgcttatttagaagtggcgcgcctcaggaaaatctgtagacaatcttg
R-Gal10-TPIpend	caaaaaacacatacataaactaaaaatgacagctcagttacaaagtgaaa
F-TPIp-Gal10end	tttcactttgtaactgagctgtcatttttagtttatgtatgtgtttttttg
R-TEFp-Gal1end	cttcttctgaatgagatttagtcattttgtaattaaaacttagattagat

F-Gal1-TEFpend	atctaatctaagttttaattacaaaatgactaaatctcattcagaagaag
R-Gal1-Hxt7tend	aaagtgtctaatgagtcagttatttttataattcatatagacagctgccc
F-Hxt7t-Gal1end	gggcagctgtctatatgaattataaaaataactgactcattagacacttt
R-Hxt7t-BamHI-pRS426end	cgaggtcgacggtatcgataagcttgatatcgaattcctgcagcccgggggatccgatcacgctctaatttgtgc
**Primers for selection and screening plasmid**	
F_Gal3prom_gene_BamHI	acgtgaggatccgcataaacaccatcagcctc
R_TEFt_NotI_LE	gactgagcggccgctggatggcggcgttagtat
Reverse Gal3_TEFt end	gttcttgaaaacaagaatctttttattgtcaccaacttgtttctttatagagtgtaagag
Forward TEFt_Gal3 end	ctcttacactctataaagaaacaagttggtgacaataaaaagattcttgttttcaagaac
F_Hxt7_BamHIend	agctcggatcctttgcgaacacttttattaatt
R_Hxt7_KanRend	catttgatgctcgatgagtttttctaataactgactcattagacactttttgaagcg
F_kanR_Hxt7end	cgcttcaaaaagtgtctaatgagtcagttattagaaaaactcatcgagcatcaaatg
Reverse KanR_Gal1p end	gtaagaatttttgaaaattcaatataaatgagccatattcaacgggaaacgtcttg
Forward Gal1p_KanR end	caagacgtttcccgttgaatatggctcatttatattgaattttcaaaaattcttac
Reverse Gal1p_GFP_ADH1t end	accagtgaataattcttcacctttagacattatagtttttctccttgacgttaaag
Forward GFP_ADH1t_Gal1p end	ctttaacgtcaaggagaaaaaactataatgtctaaaggtgaagaattattcactggt
R_ADH1t_GFP_SalIend	agatcgtcgactgccggtagaggtgtggtca
**Primers for GAL2-2.1mutagenesis and cloning in pRS405**	
For_Gal2prom_XhoIend	agttgcctcgagttgcctcaggaaggcaccggcggtc
RevGal2prom_Gal2end	gcatattgttctcctcaactgccattatgaaagaattattttttttatta
F_Gal2_Gal2promend	taataaaaaaaataattctttcataatggcagttgaggagaacaatatgc
Gal2*-F-301-314	gcagagttagatcggatcatggccggtatagaagctgaacgacgggctggcgatgcgtcc
Gal2*-R-301-314	ggacgcatcgccagcccgtcgttcagcttctataccggccatgatccgatctaactctgc
Gal2*-F-435	tcttctaaaggtgccggtaactgtacgattgtctttacctgtt
Gal2*-R-435	aacaggtaaagacaatcgtacagttaccggcacctttagaaga
Gal2*-F-50	atgaattgaaagccggtgagtcagggcctgaaggctcccaaagtgttcct
Gal2*-R-50	aggaacactttgggagccttcaggccctgactcaccggctttcaattcat
Gal2*-F-280	tgaggtgaataaggtagaagacgccaagctttccattgctaagtctaaca
Gal2*-R-280	tgttagacttagcaatggaaagcttggcgtcttctaccttattcacctca
Gal2*-F-392	tggactgtcgaaaacttggggcgtcgtaaatgtttacttttgggcgctgc
Gal2*-R-392	gcagcgcccaaaagtaaacatttacgacgccccaagttttcgacagtcca
Gal2*-F-89	cggcttcatgtttggctgggataccggtactatttctgggtttgttgtcc
Gal2*-R-89	ggacaacaaacccagaaatagtaccggtatcccagccaaacatgaagccg
Reverse Gal2_TEFt end	agaaattcgcttatttagaagtggcgcgccttattctagcatggccttgtaccacggttt
Forward TEFt_Gal2 end	aaaccgtggtacaaggccatgctagaataaggcgcgccacttctaaataagcgaatttct
Reverse TEFt_NotI end	acgtgcggccgcatctatattaccctgttatccc
**Primers for mutagenesis**	
Structure guided loop mutagenesis	
F-K93-loop1mut	aaattttagacgaaaaaaatccatccattaccttaacaaatgcggaccct
R-K93-loop1-mut	agggtccgcatttgttaaggtaatggatggatttttttcgtctaaaattt
F-S115-loop1mut	gaatggtcgaattactttaaatgcggactacatgtggcac
R-S115-loop1mut	gtgccacatgtagtccgcatttaaagtaattcgaccattc
F-K345loop2-mut	ttgaacgtttactcaagatgctacaattggtagaagaatctttctcgagg
R-K345-loop2-mut	cctcgagaaagattcttctaccaattgtagcatcttgagtaaacgttcaa
F-K381loop2-mut	ctatatcaaagagctaaacacgtttactccgaatccttaagg
R-K381-loop2-mut	ccttaaggattcggagtaaacgtgtttagctctttgatatag

Site saturation mutagenesis at A109	
For 109A mut	cctacatgnnkatagatccgtctgtgtcg
Rev 109A mut	ggatctatmnncatgtaggaaccatctaaagg
Errorprone PCR of GAL3	
F_G3gene_G3p end	gagaaaataaaagtaaaaaggtagggcaacacatagt
R_G3p_G3gene end	actatgtgttgccctaccttttacttttattttctc
Primers for synthetic shuffling	
F_Mut22	tttcgaacaaaracatttagcggttgtaga
R_Mut22	tctacaaccgctaaatgtytttgttcgaaa
F_Mut68_77	gccattagccattratrtggatatgctttgcgcagttaaaaytttagacg
R_Mut68_77	cgtctaaarttttaactgcgcaaagcatatccayatyaatggctaatggc
F_Mut_123 141	akgcggactacatgtggcacattcatacttgaaaaaaattgctccggaaagatttartaa
R_Mut_123 141	ttaytaaatctttccggagcaatttttttcaagtatgaatgtgccacatgtagtccgcmt
F_Mut_271	aatttaagagtawtagaggtaacagttg
R_Mut_271	caactgttacctctawtactcttaaatt
F_Mut_394	gtttactccgaatccwtaagggtgctta
R_Mut_394	taagcacccttawggattcggagtaaac
**Primers for cloning *Promoter-EGFP-ADH1t* constructs**	
F-Gal1p-BamHIend	acgtgaggatccacatggcattaccaccatata
F-Gal2p-BamHIend	acgtgaggatccactccaattaaatgcggtag
F-Gal4p-BamHIend	acgtgaggatcctgacggcgacacagagatg
F-Gal7p-BamHIend	acgtgaggatccgacggtagcaacaagaatat
F-Gal10p-BamHIend	acgtgaggatccatcgcttcgctgattaatta
F-Gal80p-BamHIend	acgtgaggatcctatacccctttcttctctcc
F-GCY1P-BamHIend	acgtgaggatccatggagtgtataagaattg
R-Gal1P-GFPend	tgaataattcttcacctttagacattatagttttttctccttgacgttaa
R-Gal2P-GFPend	tgaataattcttcacctttagacattatgaaagaattatttttttatta
R-Gal3P-GFPend	tgaataattcttcacctttagacatactatgtgttgccctaccttttac
R-Gal4P-GFPend	tgaataattcttcacctttagacatctttcaggaggcttgcttctctgtc
R-Gal7P-GFPend	tgaataattcttcacctttagacatttttgagggaatattcaactgt
R-Gal10P-GFPend	tgaataattcttcacctttagacatttatattgaattttcaaaaattcttac
R-Gal80P-GFPend	tgaataattcttcacctttagacatgacgggagtggaaagaacgggaaac
R-GCY1P-GFPend	tgaataattcttcacctttagacatttttctatcttaatttagtaatgag
F-GFP-Gal1Pend	ttaacgtcaaggagaaaaaactataatgtctaaaggtgaagaattattca
F-GFP-Gal2Pend	taataaaaaaaataattctttcataatgtctaaaggtgaagaattattca
F-GFP-Gal3Pend	gtaaaaaggtagggcaacacatagtatgtctaaaggtgaagaattattca
F-GFP-Gal4Pend	gacagagaagcaagcctcctgaaagatgtctaaaggtgaagaattattca
F-GFP-Gal7Pend	acagttgaatattccctcaaaaatgtctaaaggtgaagaattattca
F-GFP-Gal10Pend	gtaagaatttttgaaaattcaatataaatgtctaaaggtgaagaattattca
F-GFP-Gal80Pend	gtttcccgttctttccactcccgtcatgtctaaaggtgaagaattattca
F-GFP-GCY1Pend	ctcattactaaattaagatagaaaaatgtctaaaggtgaagaattattca
F-TEF1p-BamHIend	acgtgaggatccgccgtaccacttcaaaacac
R-TPIp-BamHIend	acgtgaggatccctacttattcccttcgagat
R-TEF1P-GFPend	tgaataattcttcacctttagacattttgtaattaaaacttagattagat
R-TPIP-GFPend	tgaataattcttcacctttagacatttttagtttatgtatgtgtttttgtagtt
F-GFP-TEF1end	atctaatctaagttttaattacaaaatgtctaaaggtgaagaattattca
F-GFP-TPIend	aactacaaaaaacacatacataaactaaaaatgtctaaaggtgaagaattattca
F-FUR4-BamHIend	agctgaggatccggatacctattcttgacatgat

F-LAP3-BamHIend	agctgaggatccagcactcatgctttcttgtaca
F-MTH1-BamHIend	agctgaggatcccactaaaacgatcagcaaatgg
F-PCL10-BamHIend	agctgaggatcccgatgacttgtacggtaaggtc
F-REE1-SacIend	agctgagagctcagatagtacaactacaaactat
R-FUR4-GFPend	accagtgaataattcttcacctttagacattattccctcctattcttattatgcgtagga
R-LAP3-GFPend	accagtgaataattcttcacctttagacattctttttaaaacaatttgtggtgctcgtaa
R-MTH1-GFPend	accagtgaataattcttcacctttagacattcctttgagtgtgtgtactctatgcgttcg
R-PCL10-GFPend	accagtgaataattcttcacctttagacattttgcaagtaatctatttgcgcatggaata
R-REE1-GFPend	accagtgaataattcttcacctttagacattgtgtttggtaatttatcttgtttgatgga
F-GFP-FUR4end	tcctacgcataataagaataggagggaataatgtctaaaggtgaagaattattcactggt
F-GFP-LAP3end	ttacgagcaccacaaattgttttaaaaagaatgtctaaaggtgaagaattattcactggt
F-GFP-MTH1end	cgaacgcatagagtacacacactcaaaggaatgtctaaaggtgaagaattattcactggt
F-GFP-PCL10end	tattccatgcgcaaatagattacttgcaaaatgtctaaaggtgaagaattattcactggt
F-GFP-REE1end	tccatcaaacaagataaattaccaaacacaatgtctaaaggtgaagaattattcactggt
**Primers for cloning XYLA*3 and XKS1 under GAL1/10 and TEF1p/TPI1p promoters**	
F_Hxt7t_BamHIend	ctagctcggatcctttgcgaacacttttattaatt
R_Hxt7_XYLA_end	tacgaggctattatcgcgatgtatcaataaaaataactgactcattagacactttttgaa
F_XYLA_Hxt7_end	ttcaaaaagtgtctaatgagtcagttatttttattgatacatcgcgataatagcctcgta
R_XYLA_Gal1p_end	aaagtaagaatttttgaaaattcaatataaatggctaaagaatatttccctcaaattcaa
R_XYLA_TEF1p_end	tagcaatctaatctaagttttaattacaaaatggctaaagaatatttccctcaaattcaa
F_Gal1p_XYLA_end	ttgaatttgagggaaatattctttagccatttatattgaattttcaaaaattcttacttt
F_TEF1p_XYLA_end	ttgaatttgagggaaatattctttagccattttgtaattaaaacttagattagattgcta
R_TEF1p_TPIp_end	atataatctcgaagggaataagtaggccgtaccacttcaaaacacccaag
F_TPIp_TEF1_end	cttgggtgttttgaagtggtacggcctacttattcccttcgagattatat
R_Gal1p_XKS1_end	tgtctgtctctgaattactgaacacaacattatagtttttctccttgacgttaaagtat
F_XKS1_Gal1p_end	atactttaacgtcaaggagaaaaaactataatgttgtgttcagtaattcagagacagaca
R_TPIp_XKS1_end	gtctctgaattactgaacacaacattttagtttatgtatgtgtttttg
F_XKS1_TPI_end	caaaaaacacatacataaactaaaaatgttgtgttcagtaattcagagac
R_XKS1_ADH1tend	agaaattcgcttatttagaagtggcgcgccttagatgagagtcttttccagttcgcttaa
F_ADH1t_XKS1end	ttaagcgaactggaaaagactctcatctaaggcgcgccacttctaaataagcgaatttct
R_ADH1t_SalIend	acgtacgtcgactgaccacacctctaccggca
**Primers for cloning GAL2-2.1 and TAL1 under GAL1/10 and TEF1p/TPI1p promoters**	
F-Hxt7t-XhoIend	ctctagctcgagtcctttgcgaacactttt
R-ADH1t-NotIend	ctgcatgcggccgctgccggtagaggtgtggtcaataaga
F-Tal1-Hxt7end	aaagtgtctaatgagtcagttatttttaagcggtaactttcttttcaatc
R-Hxt7-tal1end	gattgaaaagaaagttaccgcttaaaaataactgactcattagacacttt
F-Gal1-10P-Tal1end	gtttcttttgagctggttcagacatttatattgaattttcaaaaattctt
R-Tal1-Gal1-10Pend	aagaatttttgaaaattcaatataaatgtctgaaccagctcaaaagaaac
F-Gal2.1-Gal1-10Pend	ttaacgtcaaggagaaaaaactataatggcagttgaggagaacaatatgc
R-Gal1-10P-Gal2.1end	gcatattgttctcctcaactgccattatagtttttctccttgacgttaa
F-Tef1p-Tal1end	gtttcttttgagctggttcagacattttgtaattaaaacttagattagat
R-Tal1-TEF1pend	atctaatctaagttttaattacaaaatgtctgaaccagctcaaaagaaac
F-Gal2.1-TPIend	caaaaaacacatacataaactaaaaatggcagttgaggagaacaatatgc
R-TPIp-Gal2.1end	gcatattgttctcctcaactgccattttttagtttatgtatgtgtttttg

Supplementary Table 5: List of constants used in single and dual feedback models

Parameter	Description	Units	Values
kf83	Forward binding rate constant of Gal3p and Gal80p	$(nM.min)^{-1}$	2.5
kr83	Unbinding rate constant of Gal3p and Gal80p	$(min)^{-1}$	462
kf84	Forward binding rate constant of Gal4p and Gal80p	$(nM.min)^{-1}$	100
kr84	Unbinding rate constant of Gal4p and Gal80p	$(min)^{-1}$	1300
$\alpha G1$	Production rate of Gal3p from *GAL1p* promoter	$nM.min^{-1}$	35
$\alpha G3$	Production rate of Gal3p	$nM.min^{-1}$	8
$\alpha G4$	Production rate of Gal4p	$nM.min^{-1}$	3.6
$\alpha G80$	Production rate of Gal80p	$nM.min^{-1}$	9
$\alpha_o G80$	Basal production rate of Gal80p	$nM.min^{-1}$	5.9
KG3	Transcriptional feedback threshold of Gal3p	nM	64.9
KG80	Transcriptional feedback threshold of Gal80p	nM	1.5
n1	Hill Coefficient for Gal4p binding to *GAL1p* promoter	Dimensionless	2
n3	Hill Coefficient for Gal4p binding to *GAL3p* promoter	Dimensionless	1
n80	Hill Coefficient for Gal4p binding to *GAL80p* promoter	Dimensionless	1
γ_{G3}	Gal3p decay rate	min^{-1}	0.004
γ_{G4}	Gal4p decay rate	min^{-1}	0.0119
γ_{G80}	Gal80p decay rate	min^{-1}	0.0073
γ_{C83}	Gal3p-Gal80p complex decay rate	min^{-1}	0.0527
γ_{C84}	Gal4p-Gal80p complex decay rate	min^{-1}	0.0177

1. Karim, S. A., Curran, A. K. & Alper S. Hal. Characterization of plasmid burden and copy number in Saccharomyces cerevisiae for optimization of metabolic engineering applications. *FEMS Yeast Res.* **13,** 107–116 (2012).

2. Yano, K. & Fukasawa, T. Galactose-dependent reversible interaction of Gal3p with Gal80p in the induction pathway of Gal4p-activated genes of Saccharomyces cerevisiae. *Proc. Natl. Acad. Sci. U. S. A.* **94,** 1721–6 (1997).

3. Lavy, T., Kumar, P. R., He, H. & Joshua-Tor, L. The Gal3p transducer of the GAL regulon interacts with the Gal80p repressor in its ligand-induced closed conformation. *Genes Dev.* **26,** 294–303 (2012).

4. Gárdonyi, M., Jeppsson, M., Lidén, G., Gorwa-Grauslund, M. F. & Hahn-Hägerdal, B. Control of xylose consumption by xylose transport in recombinant Saccharomyces cerevisiae. *Biotechnol. Bioeng.* **82,** 818–24 (2003).

5. Reznicek, O. *et al.* Improved xylose uptake in Saccharomyces cerevisiae due to directed evolution of galactose permease Gal2 for sugar co-consumption. *J. Appl. Microbiol.* **119,** 99–111 (2015).

6. Reznicek, O., Sandra, F., Kassandra, L., Bernhad, S. & De Waal, P. P. Polypeptides with permease activity. (2014).

7. Ness, J. E. *et al.* Synthetic shuffling expands functional protein diversity by allowing amino acids to recombine independently. *Nat. Biotechnol.* **20,** 1251–1255 (2002).

8. Venturelli, O. S., El-samad, H. & Murray, R. M. Synergistic dual positive feedback loops established by molecular sequestration generate robust bimodal response. *Proc. Natl. Acad. Sci.* **109,** E3324–33 (2012).

9. Giniger, E. & Ptashne, M. Cooperative DNA binding of the yeast transcriptional activator GAL4. *Proc. Natl. Acad. Sci. U. S. A.* **85,** 382–386 (1988).

10. Kang, T., Martins, T. & Sadowski, I. Wild type GAL4 binds cooperatively to the GAL1-10 UASG in vitro. *J. Biol. Chem.* **268,** 9629–9635 (1993).

11. Reimand, J., Vaquerizas, J. M., Todd, A. E., Vilo, J. & Luscombe, N. M. Comprehensive reanalysis of transcription factor knockout expression data in Saccharomyces cerevisiae reveals many ne w targets. *Nucleic Acids Res.* **38,** 4768–4777 (2010).

12. Hu, Z., Killion, P. J. & Iyer, V. R. Genetic reconstruction of a functional transcriptional regulatory network. *Nat. Genet.* **39,** 683–687 (2007).

13. Ren, B. *et al.* Genome-wide location and function of DNA binding proteins. *Science (80-.).* **290,** 2306–2309 (2000).

14. Choi, I. D., Jeong, M. Y., Ham, M. S., Sung, H. C. & Yun, C. W. Novel Ree1 regulates the expression of ENO1 via the Snf1 complex pathway in Saccharomyces cerevisiae. *Biochem. Biophys. Res. Commun.* **377,** 395–399 (2008).
