# Supplementary material for: A semi-synthetic regulon enables rapid growth of yeast on xylose
Source: Nat Commun. 2018 Mar 26;9:1233. doi: 10.1038/s41467-018-03645-7 (PMC5964326; doi:10.1038/s41467-018-03645-7)
Supplement: Supplementary file 3 — Description of Additional Supplementary Files(PDF 170 kb) [file 41467_2018_3645_MOESM3_ESM.pdf]

Description of Additional Supplementary Files

File Name: Supplementary Data 1
Description: **Differential gene expression analysis directly due to Gal4p.** Gal4p controlled genes from YEASTRACT that are differentially expressed in REG strains.

File Name: Supplementary Data 2
Description: **Differentially expressed transcription factors.** Genes that code for transcription factors (TFs) from YEASTRACT that are differentially expressed in REG strains.
