# Supplementary material for: A semi-synthetic regulon enables rapid growth of yeast on xylose
Source: Nat Commun. 2018 Mar 26;9:1233. doi: 10.1038/s41467-018-03645-7 (PMC5964326; doi:10.1038/s41467-018-03645-7)
Supplement: Supplementary file 4 — Supplementary Data 1(PDF 110 kb) [file 41467_2018_3645_MOESM4_ESM.pdf]

GAL4 controlled genes from YEASTRACT that are differentially experessed in REG strains

AAT2
ACS2
ADH1
AFR1
AIM2
AMD1
ATP16
ATP2
ATR1
BAR1
BUB2
CAF120
CHS7
CIR1
CIS3
CIT3
CLN3
CMC1
CNL1
CPA1
CTS1
CWC25
CWP1
CYC3
DAP1
DED1
DFG16
DMA1
DMA2
DPL1
DPS1
DSE2
DUG1
ECM33
EGT2
EMP46
ERG4
ERV46
EXG2
FAA1
FRQ1
FUM1
FUN26
GAS1
GAS3
GCY1

GGC1
GIC2
GIP2
GSH1
HEK2
HEM2
HFD1
HHF2
HMS2
HNT3
HOG1
HOM2
HOM6
HSP10
HTA2
HTB1
HXT1
HXT2
IMG2
IPT1
ISY1
LAC1
LTP1
LYP1
LYS1
MBR1
MCH5
MDH1
MEF1
MET17
MF(ALPHA)1
MIP1
MIR1
MRP17
MRP21
MRP4
MRPL24
MRPL31
MRPL32
MRPL37
MRPL38
MRPL4
MRPS16
MRPS17
MRPS18
MSK1
MSS51

MTM1
MUM2
MYO1
MZM1
NPL3
NTE1
NTH2
NTR2
OPT2
OSW5
OXA1
PAA1
PCL10
PCS60
PET9
PHO80
PHS1
PIR3
PLB2
PLB3
PMA2
PMT2
POR1
POS5
PST1
PTA1
PTR2
QDR1
RBD2
RDR1
RET2
RIB4
RIO1
RNR4
RPL3
RPS22B
RSM27
RTT102
RUP1
SAM1
SAM2
SCT1
SCW10
SCW11
SCW4
SEO1
SFB2

SFL1
SHY1
SLS1
SNQ2
SRL1
SSC1
SSU1
SUN4
SUR7
SWS2
TDA7
TIM13
TIP1
TOA1
TOM40
TRX1
TUB2
USA1
USO1
UTH1
VMA5
VPH1
WHI3
WSC2
WTM1
YBL029W
YBR197C
YCR023C
YDR010C
YER152C
YHB1
YKL031W
YLR030W
YME1
YMR147W
YOR283W
YOX1
YPL067C
YPL071C
YPR053C
ZEO1
