# Supplementary material for: A semi-synthetic regulon enables rapid growth of yeast on xylose
Source: Nat Commun. 2018 Mar 26;9:1233. doi: 10.1038/s41467-018-03645-7 (PMC5964326; doi:10.1038/s41467-018-03645-7)
Supplement: Supplementary file 5 — Supplementary Data 2(PDF 98 kb) [file 41467_2018_3645_MOESM5_ESM.pdf]

Genes that code for transcription factors (TFs) from YEASTRACT that are differentially experessed in REG stra

Aft1
Aro80
Cbf1
Cup9
Eds1
Gcn4
Gsm1
Haa1
Hcm1
Ino4
Mig3
Pdr3
Rdr1
Rds2
Rph1
Rsf2
Sfl1
Stb5
Stp2
Sum1
Sut2
Swi5
Tye7
Vhr1
War1
YKL222C
YNR063W
Yap3
Yox1

ins
